# Identifying Predictors of Activity Based Anorexia Susceptibility in Diverse Genetic Rodent Populations

**DOI:** 10.1371/journal.pone.0050453

**Published:** 2012-11-30

**Authors:** Eneda Pjetri, Ria de Haas, Simone de Jong, Cigdem Gelegen, Hugo Oppelaar, Linda A. W. Verhagen, Marinus J. C. Eijkemans, Roger A. Adan, Berend Olivier, Martien J. Kas

**Affiliations:** 1 Rudolf Magnus Institute of Neuroscience, Department of Neuroscience and Pharmacology, University Medical Centre Utrecht, Utrecht, The Netherlands; 2 Center for Neurobehavioral Genetics, University of California Los Angeles, Los Angeles, California, United States of America; 3 Department of Biophysics, Imperial College London, London, United Kingdom; 4 Institute for Genetics, Department of Mouse Genetics and Metabolism, University of Cologne, Cologne, Germany; 5 Julius Center for Health Sciences and Primary Care, University Medical Center Utrecht, Utrecht, The Netherlands; 6 Utrecht Institute for Pharmaceutical Sciences, Division of Pharmacology, Faculty of Science, Utrecht University, Utrecht, The Netherlands; 7 Department of Psychiatry, Yale University School of Medicine, New Haven, Connecticut, United States of America; University of Chicago, United States of America

## Abstract

Animal studies are very useful in detection of early disease indicators and in unravelling the pathophysiological processes underlying core psychiatric disorder phenotypes. Early indicators are critical for preventive and efficient treatment of progressive psychiatric disorders like anorexia nervosa. Comparable to physical hyperactivity observed in anorexia nervosa patients, in the activity-based anorexia rodent model, mice and rats express paradoxical high voluntary wheel running activity levels when food restricted. Eleven inbred mouse strains and outbred Wistar WU rats were exposed to the activity-based anorexia model in search of identifying susceptibility predictors. Body weight, food intake and wheel running activity levels of each individual mouse and rat were measured. Mouse strains and rats with high wheel running activity levels during food restriction exhibited accelerated body weight loss. Linear mixed models for repeated measures analysis showed that baseline wheel running activity levels preceding the scheduled food restriction phase strongly predicted activity-based anorexia susceptibility (mice: Beta  =  −0.0158 (±0.003 SE), P<0.0001; rats: Beta  =  −0.0242 (±0.004 SE), P<0.0001) compared to other baseline parameters. These results suggest that physical activity levels play an important role in activity-based anorexia susceptibility in different rodent species with genetically diverse background. These findings support previous retrospective studies on physical activity levels in anorexia nervosa patients and indicate that pre-morbid physical activity levels could reflect an early indicator for disease severity.

## Introduction

Eating disorders are severe psychiatric illnesses with high morbidity and mortality. Particularly anorexia nervosa (AN) has the highest mortality rate among psychiatric disorders [1], [2]. AN affects young females and has an incidence rate of around 0.9% [3]. Hallmark of the illness is a low a body weight and the refusal to maintain a normal body weight [4]. Various risk factors have been identified and genetics is found to play a role in the development of AN [5], [6], [7], [8], [9], [10]. However, little is known about aetiology or predictive factors of this disease. Identifying predictive factors of specific AN phenotypes, such as physical hyperactivity [11], [12], could improve the treatment approach and increase treatment efficacy.

Physical activity levels are important in AN; they play a role in the onset and maintenance of illness [13], [14], [15] and have an influence on the recovery rate [16], [17], [18]. High activity levels may precede dieting [19], suggesting that pre-morbid physical activity may be a predictor of illness course during times of reduced food intake. During the illness, regardless of low body weight, an increase in physical activity is observed [12], [20]. The effects of limited food intake and hyperactivity under these conditions can be modelled in animals [21], [22].

Animal studies take place under controlled genetic and environmental conditions, minimizing their complex interaction effects on phenotypic heterogeneity. They may provide novel insights in pre-morbid factors that can affect and/or predict the course of the disorder. This is a challenge in human studies, as currently the research on these factors is determined either via cross-sectional or retrospective studies. Longitudinal prospective studies are very sparse (e.g. Nicholls & Viner [23]) and face the difficulty of low incidence rate. Activity based anorexia (ABA) is an animal model of pathophysiological processes of AN where the combined effects of daily scheduled limited food availability and voluntary running wheel activity mimic the physical hyperactivity behaviour observed in AN patients while their food intake is severely reduced. In this model, rodents have unlimited voluntary access to a running wheel throughout the experiment, while food is *ad libitum* available for a limited period at the same time during several consecutive days [21], [22]. The resulting physical hyperactivity under this conditions is a core feature of this model [24], [25] and has clinical relevance [26], [27].

Previous studies performed in our laboratory mapped the hyperactivity behaviour in chromosome substitution (CS) strain mice and has identified CS strains becoming hyperactive or being susceptible to this model [28]. Following these results, we continued our research focusing on the question: which factors could influence and/or predict susceptibility to the model? By using this animal model, we investigated the response of 11 different inbred mice strains and a set of outbred Wistar WU rats, to daily scheduled food restriction and studied the relationship between their baseline phenotypes and their ABA susceptibility.

## Materials and Methods

### Ethics Statement

All animal experiments were approved by the Animal Experimentation Committee of University Medical Centre Utrecht and were carried out in agreement with the Dutch Law (Wet op de Dierproeven, 1996) and European regulations (Guideline 86/609/EEC).

### Animals and housing

Initial breeding pairs of the inbred mouse strains were obtained from The Jackson Laboratory (Bar Harbor, ME, USA). The mice used in the experiment were bred at the Rudolf Magnus Institute of Neuroscience animal facility. The following strains were tested: A/J (n = 8) (JAX stock # 000646), AKR/J (AKR n = 8) (JAX stock #000648), BALB/cByJ (BALB n = 7) (JAX stock # 001026), C3H/HeJ (C3H n = 12) (JAX stock # 000659), C57BL/6J (B6J n = 10) (JAX stock # 000664), CAST/EiJ (CAST n = 8) (JAX stock # 000928), DBA/2J (DBA n = 7) (JAX stock # 000671), FVB/NJ (FVB n = 8) (JAX stock # 001800), KK/HlJ (KK n = 10) (JAX stock # 002106), NZW/LacJ (NZW n = 10) (JAX stock # 001058) and WSB/EiJ (WSB n = 10) (JAX stock # 001145). The selected strains are part of Mouse Phenome Database (MPD) priority strains, Tier 1 (http://phenome.jax.org/db/q?rtn=strains/search&reqpanel=MPD). Following weaning at 3–4 weeks, female and male mice were separately housed in groups (2–5 mice per cage) in cages (Macrolon ®, Tecniplast, Milan, Italy). Outbred Wistar WU rats (n = 34) were obtained from Harlan (Horst, The Netherlands) and were individually housed upon arrival. The housing facilities were maintained on a 12:12 h dark/light cycle with an ambient temperature of 21.0±2°C and humidity of 55±10%. During this period, the mice and rats were given water and food *ad libitum* (Rat and Mouse Breeder and Grower, Special Diet Services, Essex, England). For this study we used test-naive 3–4 months old female mice and female rats, because of the high prevalence of AN in females [2].

### Surgical procedures for rats

One week after arrival, all rats received transmitters (TA10TA-F40; Data Sciences International, St Paul, Minnesota, USA) in the abdominal cavity under fentanyl–fluanisone (0.1 mL/100 g body weight, i.m.; Hypnorm, Janssen Pharmaceutica, Beerse, Belgium) and midazolam (0.05 mL/100 g body weight, i.p.; Dormicum, Hoffman-LaRoche, Mijdrecht, The Netherlands) anaesthesia. After surgery, rats were treated with carprofen (0.01 mL/100 g body weight, s.c.; Rimadyl, Pfizer Animal Health, Capelle a/d Ijssel, The Netherlands) and saline (3 mL, s.c.) and allowed to recover for 2 weeks. With these transmitters, the locomotor activity, while not in the wheel, was measured continuously.

### Experimental procedure

After an adaptation phase in individual cages where the rodents had *ad libitum* access to food, water and running wheel, they were exposed to a daily restricted feeding schedule (restriction phase); mice for 4 consecutive days and rats for 5 consecutive days. The adaptation phase was 7 days for mice and 10 days for rats; from our previous experiments it was observed that rats need few more days to adapt to the presence of a wheel. During this period, food (for mice 5 pellets and for rats 12 pellets of about 1.2 g each with an energy intake of 3.31 kilocalorie (kcal) per gram) was available only in the beginning of the dark phase for 2 hrs for the mice and 1.5 hrs for the rats.

Baseline measures for body weight and food intake were generated on the basis of the average value during the last three days of the adaptation phase (measured just before the dark period). During these days, the animals were left undisturbed until the start of the restriction phase. Baseline wheel running activity (wheel revolutions) (WRA) levels were determined as the average WRA of the last two days of the adaptation phase, just before the restriction phase, when the activity levels are stable. WRA levels were measured daily during the restriction phase.

The transmitters implanted in the rats were switched on by magnetic field induction during the baseline phase of the experiment. Baseline locomotor activity levels (LMA) were determined as the average LMA of the last two days of the adaptation phase. The recording was continuous during the restriction days. For a group of rats (n = 6) because of a technical malfunction, there were no LMA recordings for days 2 and 3 of the restriction phase.

The level of body weight loss during the scheduled food restriction days was selected as a measure of susceptibility to the ABA. In mice, if a mouse lost 15% or more of their baseline BW on any experimental day, that mouse was taken out of the experiment (based on the ethical humane endpoint criteria) and was considered susceptible to ABA. In rats, if a rat lost 25% or more of their baseline body weight on any experimental day, that rat was taken out of the experiment (based on the ethical humane endpoint criteria) and was considered susceptible to ABA. Based on the level of body weight loss, a second dichotomous variable for ABA susceptibility was generated, with each individual mouse and rat assigned a value whether it was susceptible or not. In this way, a percentage of mice within each strain and a percentage of rats that were susceptible to activity based anorexia model (ABA) i.e. that could not maintain the body weight above 85% (mice) and 75% (rats) of their baseline body weight, was determined.

### Statistical analysis

The data were expressed as means with standard error of the mean (SEM) unless otherwise specified. One sample Kolmogorov-Smirnov test was used to check the Gaussian distribution and Levene's test was used for the homogeneity of variances.

To assess the effects of the baseline parameters (body weight, food intake, activity and food intake per body weight, and in rats also locomotor activity levels based on telemetry data) as predictive factors, linear mixed models for repeated measures analysis, which takes into account the longitudinal data and within-mouse correlations, were used. To analyze the susceptibility to the model, body weight loss during the restriction phase was used as the dependent variable. Animal's identification number was entered as random effects, while in the fixed effects, the days of the experiment and each of the baseline factors were entered. To check whether there was a specific mouse inbred strain that contributed to the effect of these factors, the analysis was repeated with each of the mouse inbred strains being taken out of the analysis in turns.

Two-sided P values of <0.05 were considered statistically significant. The effects were expressed as standardized beta (Beta) with standard error (SE), denoting the change in dependent variable with one standard deviation change of the independent variable. Spearman's coefficient of rank correlation was used for correlation analysis as the data were not normally distributed. Significance was set at P<0.05.

Statistical analysis was carried out using SPSS 15.0 for Windows (SPSS, Chicago, IL, USA).

## Results

### Baseline parameters

#### Inbred mouse strains

**Figure 1:**
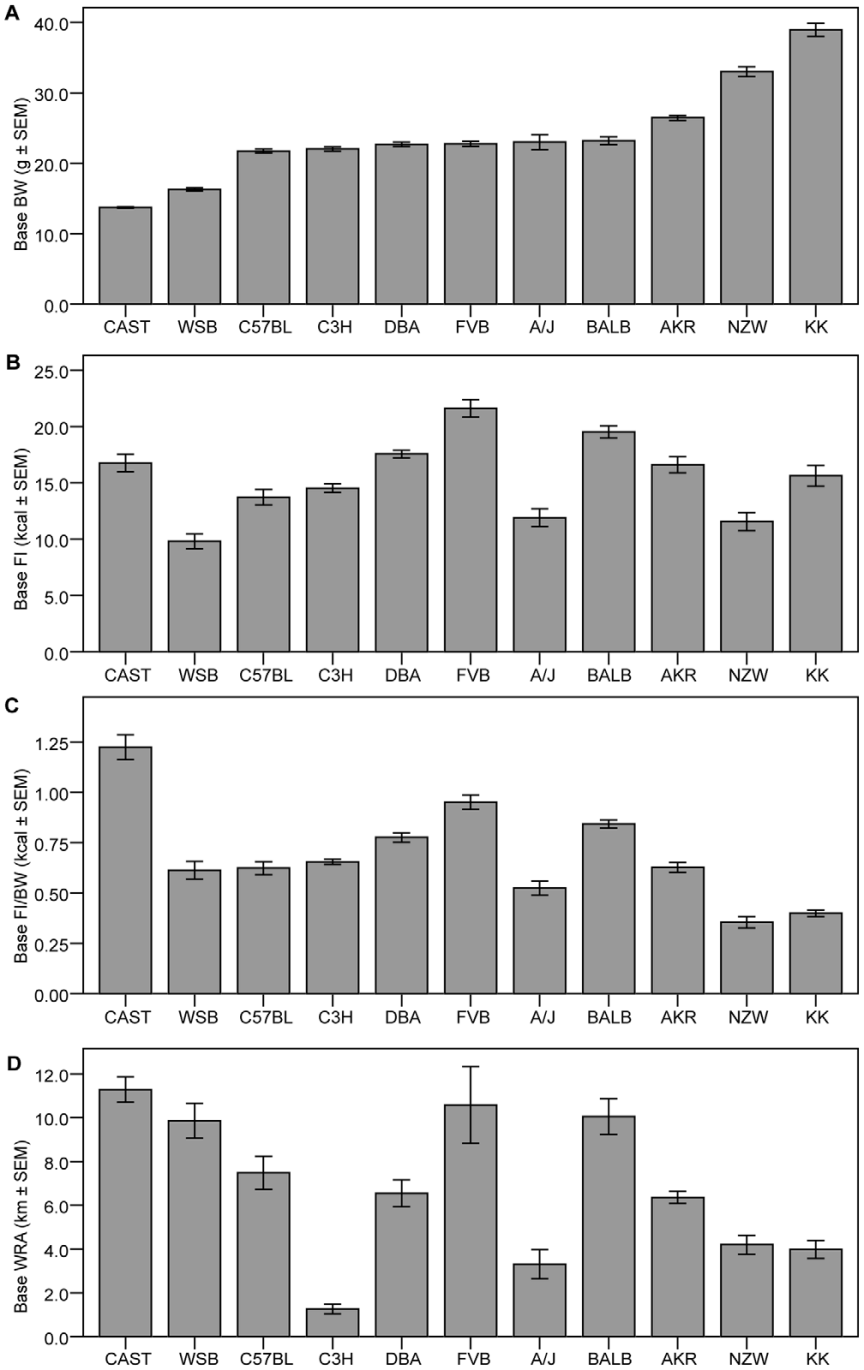
Baseline (Base) body weight (BW) (A), food intake (FI) (B), food intake corrected per body weight (FI/BW) (C) and wheel running activity (WRA) levels (D). The data are presented as mean ± SEM. The ranking for all graphs in this panel is based on baseline body weight.

There was a wide distribution of baseline body weight between strains. However, the majority of the strains had a baseline body weight (BW) between 20 g and 26 g. CAST and WSB mice had the lowest BW with 13.72 g (±0.09 SEM) and 16.27 g (±0.24 SEM), respectively. On the other side of the baseline body weight spectrum are NZW and KK strains with a BW of 32.99 g (±0.71 SEM) and 38.94g (±0.92 SEM), respectively (figure 1A). Food intake (FI) among strains varied, with FVB strain having the highest baseline FI 21.60kcal (±0.77 SEM) followed closely by BALB strain 19.51kcal (±0.53 SEM; figure 1B). When individual levels of food intake were corrected for individual levels of body weight, CAST strain had the highest food intake per body weight (FI/BW) levels (1.22kcal ±0.06 SEM); followed by FVB strain (0.95kcal ±0.04 SEM; figure 1C). Baseline WRA levels ranged from approx. 750 to 35000 revolutions per day, corresponding to 0.33 and 15.58 kilometres (km) per day, respectively (figure 1D).

#### Wistar WU rats

The rats' baseline body weight ranged between 194 and 253 g, and their food intake was between 55.38 to 81.33kcal. Food intake corrected for body weight was between 0.25 to 0.37kcal (data not shown). Baseline WRA levels were from approx. 270 to 13400 corresponding to 0.3 to 13.4 km. Non-specific locomotor activity levels (measured via the telemetry system) were between approx. 525 and 6500 counts (data not shown).

### Predictive factors for ABA susceptibility

#### Inbred mouse strains

**Figure 2:**
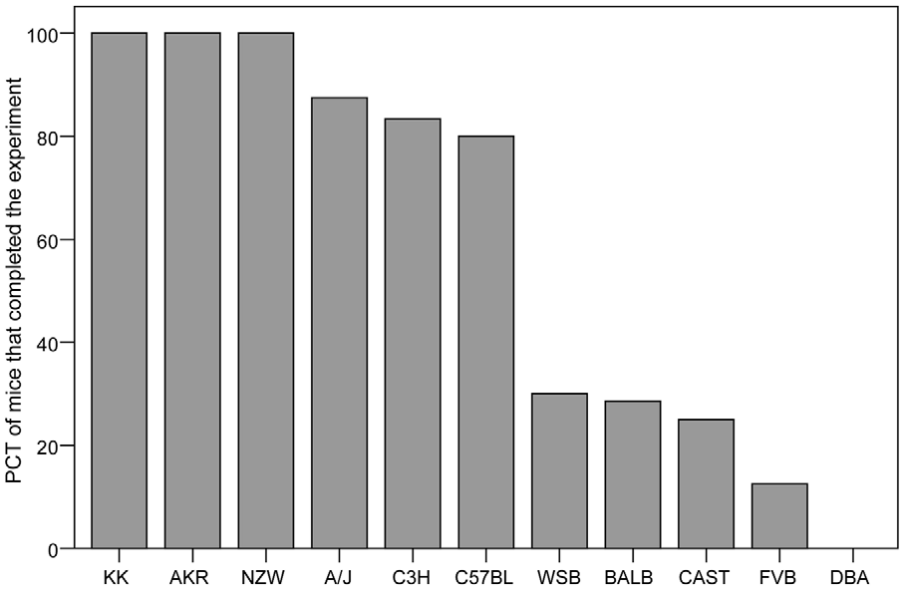
Percentage (PCT) of mice within each strain that was susceptible to activity based anorexia model (ABA) i.e. that could not maintain the body weight above 85% of their baseline body weight.

Nearly half of the strains were particularly susceptible in this model. Within these strains, less than 25% of the mice remained at a BW level above 85% of their baseline BW until the end of the experimental days (figure 2).

Linear mixed models for repeated measures analysis revealed that among baseline factors (BW, FI and WRA), WRA levels had the strongest effect on ABA susceptibility (body weight loss), based on Beta estimate (Beta  =  −0.0158 (±0.003 SE), P<0.0001). BW was the second factor (Beta  = 0.0118 (±0.003 SE), P = 0.0005) and FI was third (Beta  =  −0.0102 (±0.004 SE), P = 0.0057). The derived baseline FI/BW had also a significant effect (Beta  =  −0.0106 (±0.004 SE), P = 0.0041). The interaction of the derived FI/BW and baseline WRA levels (FBxW) had also a strong effect on ABA susceptibility, where the effect of WRA levels was increased with the effect of FI/BW (Beta  = 0.0199 (±0.004 SE), P<0.0001).

The high predictive value of baseline WRA levels for ABA susceptibility was not dependent on a specific genetic background, as was revealed by a repeated single strain exclusion analysis (data not shown). Based on this additional analysis, FI became an non-significant predictor when FVB strain was taken out (Beta  =  −0.0053 (±0.004 SE), P = 0.164). Similarly, when NZW strain was taken out, FI/BW had no longer a significant effect (Beta  =  −0.0066 (±0.004 SE), P = 0.0810). Further, also the FBxW interaction effect was also driven by one strain, CAST strain. When CAST strain was taken out of the analysis FBxW interaction effect was non-significant (Beta  = 0.0059 (±0.003 SE), P = 0.080), indicating that these specific strains dominated the effects of these measures.

#### Wistar WU rats

In the five days of daily scheduled food restriction Wistar WU rats lost weight, however, none of them reached the humane end point criteria (25% body weight loss). Linear mixed models for repeated measures analysis showed that only physical activity measures, WRA and LMA levels, had a significant effect on the level of body weight loss. Among the two, WRA levels had the strongest effect (WRA Beta  =  −0.0242 (±0.004 SE), P<0.0001; LMA Beta  =  −0.0151 (±0.005 SE), P = 0.005). The derived baseline FI/BW had no effect on body weight loss and there was no interaction with baseline WRA or baseline LMA levels.

### Correlations

#### Inbred mouse strains

**Figure 3:**
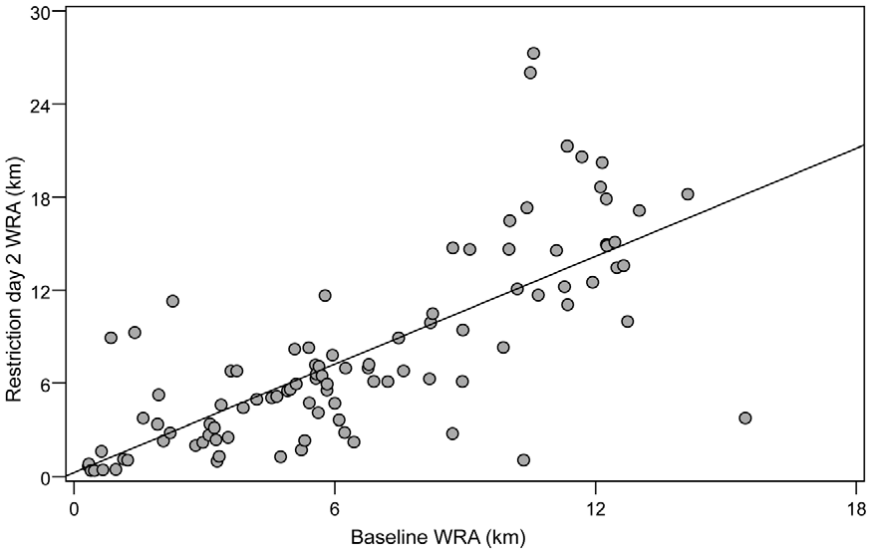
Significant correlation between baseline wheel running activity (WRA) levels (x-axis) and restriction day 2 WRA levels (y-axis) across individual mice (n = 98) from 11 different mouse inbred strains (r_s_  = 0.767, P<0.0001).

**Figure 4:**
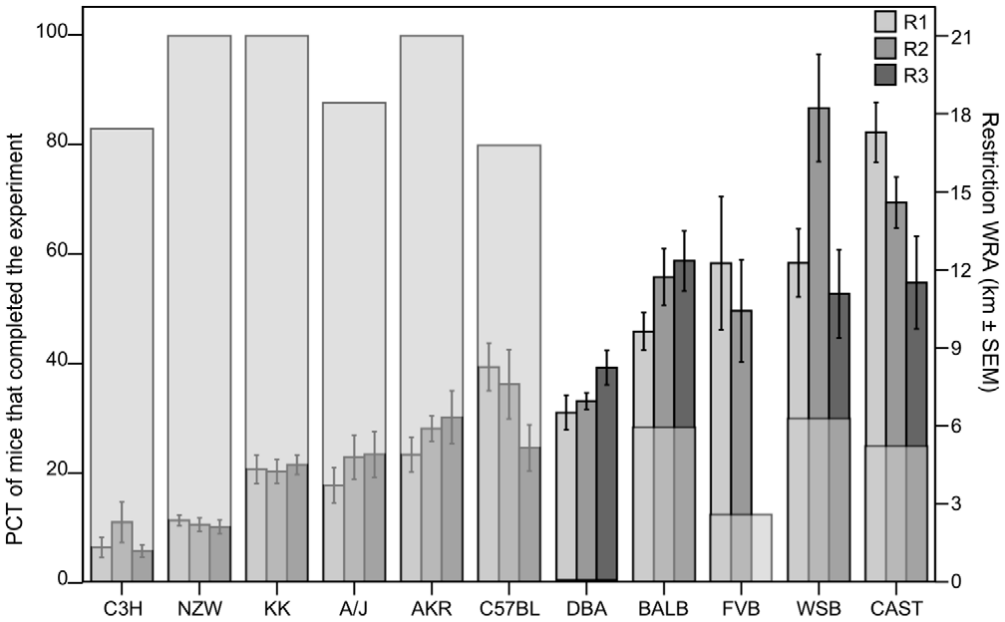
Overlay between percentage (PCT) of mice within each strain that completed the experiment, left y-axis (transparent gray bars), and restriction wheel running activity (WRA) levels on day 1 (R1), day 2 (R2) and day 3 (R3) right y-axis (ranking based on the right y-axis); in 11 different mouse inbred strains, x-axis. Please note that because on R3, more than 80% of FVB strain mice were taken out of the experiment, the data for the remaining mice on this day (n = 2) are not shown. Strains with overall high activity levels and strains that increased their activity during the restriction days (e.g. DBA), are susceptible to the model.

Based on all individual values, baseline BW was not correlated with baseline FI, but was weakly and negatively correlated to baseline WRA levels (r_s_  =  −0.380, P<0.001; data not shown). Baseline FI was also weakly correlated to baseline WRA levels, but in a positive direction (r_s_  = 0.418, P<0.001; data not shown). There was a positive correlation between baseline WRA levels and FI/BW (r_s_  = 0.609, P<0.0001). Baseline WRA and WRA levels on restrictive days (R) 1, 2, 3 and 4 were strongly positively correlated to each other (R1 (n = 98) r_s_  = 0.885, P<0.0001; R2 (n = 98) r_s_  = 0.767, P<0.0001; R3 (n = 78) r_s_  = 0.768, P<0.0001; R4 (n = 64) r_s_  = 0.593, P<0.0001). Figure 3 shows the correlation of baseline and restriction days 2 (R2) WRA levels, where all the mice are still in the experiment. Restriction days WRA levels were also negatively correlated ABA susceptibility (dichotomous variable) (R1 (n = 98) (r_s_  =  −0.575, P = <0.0001; R2 (n = 98) r_s_  =  −0.573, P = <0.0001; R3 (n = 78) r_s_  =  −0.360, P = 0.001; R4 (n  = 64) r_s_  =  −0.365, P = 0.003; overlay figure 4).

#### Wistar WU rats

Unlike inbred mouse strains, there was a significant but weak correlation between baseline BW and baseline FI (r = 0.425, P = 0.012). Nor FI either FI/BW were correlated to WRA levels or to LMA levels (data not shown). Baseline BW was also not correlated to WRA levels or to LMA levels (data not shown). There was a positive correlation between baseline levels of WRA and LMA (r_s_  = 0.651, P<0.0001).

Baseline LMA levels were weakly correlated to R1 LMA levels (r_s_  = 0.421, P = 0.013) but were strongly correlated to the other restrictive LMA levels (R2 r_s_  = 0.742, P<0.0001; R3 r_s_  = 0.708, P<0.0001; R4 r_s_  = 0.820, P<0.0001; data not shown).

Baseline WRA levels, though not significantly correlated to R1 WRA levels (r = 0.323, P = 0.062) were strongly correlated to the other restrictive days WRA levels (R2 r = 0.725, P<0.0001; R3 r = 0.815, P<0.0001; R4 r = 0.827, P<0.0001; data not shown).

## Discussion

In the present study, using eleven inbred mouse strains, we found that baseline WRA (physical activity) level was the strongest factor to predict ABA susceptibility when compared to other baseline factors, such as food intake and body weight. In rats, baseline WRA and LMA (physical activity) levels were the only factors to influence body weight loss. These findings support the notion from retrospective studies in human AN indicating that physical hyperactivity levels may precede anorexia nervosa onset and can also predict the physical activity levels during AN illness [14], [19], [29]. These findings provide novel opportunities to study neurobiological mechanisms underlying this potential clinical relevant trait. Genetic studies may be a good starting point, as initial genetic factors for general daily physical activity levels have been identified in animals [30], [31], [32], [33], [34] and in humans [35], [36]. Genetic mapping studies in different genetic reference populations have found a genetic locus on mouse chromosome 1 related to activity [37], [38]. Furthermore, studies performed in our laboratory have identified mouse chromosomes that contribute to hyperactivity under scheduled food restriction conditions [28]. Next, specific genes coupled to specific components of physical hyperactivity levels during limited food access [39]. Understanding the neurobiological contributions of these genes to these behavioural phenotypes may be a critical step forward in the development of aetiology – directed treatment.

In addition, the interaction between physical activity levels and food intake per body weight had initially a substantial effect on ABA susceptibility in the inbred mouse strains. However, further analysis revealed that this effect was mainly driven by the CAST mouse strain which had the highest food intake per body weight levels when compared to the other strains, and was also one of the strains with the highest baseline activity levels. Similarly, the effect of baseline food intake and highest food intake per body weight became non-significant when FVB and NZW strains were taken out of the analysis, respectively. Interestingly, FVB strain had the highest FI levels among the inbred strains and was also one of the strains with high baseline activity levels, while NZW strain had the lowest FI/BW levels. These data suggests that individual mice with high baseline food intake per body weight and high innate physical activity levels have more difficulty to maintain body weight during scheduled food availability than mice that were less active or had lower food intake per body weight under baseline conditions. Translating these mouse findings to human behaviour, they would suggest that patients with high pre-morbid activity levels and high energy requirements are prone to lose weight at a higher rate in the development of illness. Subsequently, with illness progression, the basal metabolic rate decreases and the amount of energy spent with any activity is less than that in normal controls [40]. Nevertheless, the physical activity levels do affect the rate of recovery [16], [17], [18] indicating the importance of this trait as a predictive factor as well as a factor affecting disease maintenance.

The other baseline factors, such as body weight and food intake, and their derivative food intake per body weight, also exhibited a significant predictor effect on ABA susceptibility, in inbred mouse strains but not in Wistar WU rats. However, in the inbred strains, the magnitude of their predictive value was lower than that of baseline physical activity levels. A closer look at body composition data available in the existing literature, for inbred mouse strains, showed that body fat percentage was similar to the body weight range. The strains with high body weight had also the highest fat percentage, while the rest of the strains had a similar percentage around 20% [41]. These data, even though it was not possible to directly statistically test their effect on ABA susceptibility, suggest that body composition effect could be similar to that of body weight with a low magnitude effect. Nonetheless, as pre-morbid BW levels have been previously correlated in AN with the BW at referral [42] and also at follow up [43] this may still be an important predictive factor to consider. Interestingly, the relationship between baseline body weight and ABA susceptibility was strong in some of the mouse inbred strains tested and not present in others (data not shown), suggesting that this could be independent of their body composition (they were different in body weight and fat percentages) and their levels of prediction may depend on other factors related to their genetic background.

Although fat percentages were not very different between the strains, circulating leptin levels could be different as leptin release is associated to the fat cell size. Therefore, we were also interested if strain differences in leptin signalling may be related to physical activity levels. While the leptin data for the eleven inbred mouse strains are available only after a fat diet for several weeks [44], the strain ranking pattern for leptin signalling was very different from that of the strain ranking for physical activity levels in the present study. Other metabolic factors such as insulin levels (after fat diet) or glucose levels (baseline or after fat diet) [44] again did not show a specific pattern that could be correlated to ABA susceptibility. These data indicate that none of these metabolic factors is a common factor that could explain the ABA susceptibility in these inbred mouse strains.

The difference that we observe between the outbred rats and inbred mouse strains in ABA susceptibility levels could be attributed to the humane end point criteria, which is reached sooner in mice (15% body weight loss) than in rats (25% body weight loss).

Yet, Wistar WU rats lost weight during the restriction days and apart from baseline physical activity levels, the other baseline parameters did not have a significant effect on this body weight loss. The derived FI/BW also did not have an effect and there was no interaction with physical activity data. Interestingly, the present study reveals that the relationship between baseline physical activity levels and level of body weight loss in the ABA model is observed in different rodent species (rats and mice).

Taken together, physical activity levels strongly predict ABA susceptibility. The effect of physical activity levels on ABA susceptibility in individual inbred mouse strains is dependent on the genetic background. Baseline body weight and food intake are in general less strong predictors of ABA susceptibility in inbred mouse strains. The effect of body weight and food intake on ABA susceptibility is sometimes strongly dominated by a single strain (e.g. CAST mouse strain effect on the interaction between physical activity levels and food intake per body weight and FVB mouse strain effect on food intake). Literature search revealed no common metabolic factor that could contribute to body weight loss in inbred strains. While further studies are necessary to unravel the neurobiological mechanisms of these factors and their interrelatedness, these findings and the retrospective AN studies on physical activity levels suggest that pre-morbid physical activity levels of each patient could already be taken into consideration as a possible predictor of illness severity.

## References

[1] Arcelus J, Mitchell AJ, Wales J, Nielsen S (2011) Mortality rates in patients with anorexia nervosa and other eating disorders. A meta-analysis of 36 studies. Arch Gen Psychiatry 68: 724–731. 21727255 10.1001/archgenpsychiatry.2011.74

[2] Hoek HW (2006) Incidence, prevalence and mortality of anorexia nervosa and other eating disorders. Curr Opin Psychiatry 19: 389–394. 16721169 10.1097/01.yco.0000228759.95237.78

[3] Hudson JI, Hiripi E, Pope HG Jr, Kessler RC (2007) The prevalence and correlates of eating disorders in the National Comorbidity Survey Replication. Biol Psychiatry 61: 348–358. 16815322 10.1016/j.biopsych.2006.03.040 PMC1892232

[4] APA (1994) Diagnostic and Statistical Manual of Mental Disorders Washington DC.: American Psychiatric Association 886 pages p.

[5] Bulik CM, Thornton LM, Root TL, Pisetsky EM, Lichtenstein P, et al (2010) Understanding the relation between anorexia nervosa and bulimia nervosa in a Swedish national twin sample. Biol Psychiatry 67: 71–77. 19828139 10.1016/j.biopsych.2009.08.010 PMC2851013

[6] Herzog DB, Eddy KT (2009) Eating disorders: what are the risks? J Am Acad Child Adolesc Psychiatry 48: 782–783. 19628994 10.1097/CHI.0b013e3181aa03d7 PMC2742997

[7] Jacobi C, Hayward C, de Zwaan M, Kraemer HC, Agras WS (2004) Coming to terms with risk factors for eating disorders: application of risk terminology and suggestions for a general taxonomy. Psychol Bull 130: 19–65. 14717649 10.1037/0033-2909.130.1.19

[8] Mazzeo SE, Bulik CM (2009) Environmental and genetic risk factors for eating disorders: what the clinician needs to know. Child Adolesc Psychiatr Clin N Am 18: 67–82. 19014858 10.1016/j.chc.2008.07.003 PMC2719561

[9] Pike KM, Hilbert A, Wilfley DE, Fairburn CG, Dohm FA, et al (2008) Toward an understanding of risk factors for anorexia nervosa: a case-control study. Psychol Med 38: 1443–1453. 18070371 10.1017/S0033291707002310 PMC2669537

[10] Stice E (2002) Risk and maintenance factors for eating pathology: a meta-analytic review. Psychol Bull 128: 825–848. 12206196 10.1037/0033-2909.128.5.825

[11] Casper RC (1998) Behavioral activation and lack of concern, core symptoms of anorexia nervosa? Int J Eat Disord 24: 381–393. 9813763 10.1002/(sici)1098-108x(199812)24:4<381::aid-eat5>3.0.co;2-q

[12] Kron L, Katz JL, Gorzynski G, Weiner H (1978) Hyperactivity in anorexia nervosa: a fundamental clinical feature. Compr Psychiatry 19: 433–440. 679677 10.1016/0010-440x(78)90072-x

[13] Dalle Grave R, Calugi S, Marchesini G (2008) Compulsive exercise to control shape or weight in eating disorders: prevalence, associated features, and treatment outcome. Compr Psychiatry 49: 346–352. 18555054 10.1016/j.comppsych.2007.12.007

[14] Davis C, Blackmore E, Katzman DK, Fox J (2005) Female adolescents with anorexia nervosa and their parents: a case-control study of exercise attitudes and behaviours. Psychol Med 35: 377–386. 15841873 10.1017/s0033291704003447

[15] Davis C, Kennedy SH, Ravelski E, Dionne M (1994) The role of physical activity in the development and maintenance of eating disorders. Psychol Med 24: 957–967. 7892363 10.1017/s0033291700029044

[16] Kaye WH, Gwirtsman HE, Obarzanek E, George DT (1988) Relative importance of calorie intake needed to gain weight and level of physical activity in anorexia nervosa. Am J Clin Nutr 47: 989–994. 3376913 10.1093/ajcn/47.6.989

[17] Solenberger SE (2001) Exercise and eating disorders: a 3-year inpatient hospital record analysis. Eat Behav 2: 151–168. 15001043 10.1016/s1471-0153(01)00026-5

[18] Bratland-Sanda S, Sundgot-Borgen J, Rø Ø, Rosenvinge JH, Hoffart A, et al (2010) Physical activity and exercise dependence during inpatient treatment of longstanding eating disorders: An exploratory study of excessive and non-excessive exercisers. International Journal of Eating Disorders 43: 266–273. 19839057 10.1002/eat.20769

[19] Davis C, Katzman DK, Kaptein S, Kirsh C, Brewer H, et al (1997) The prevalence of high-level exercise in the eating disorders: etiological implications. Compr Psychiatry 38: 321–326. 9406737 10.1016/s0010-440x(97)90927-5

[20] Casper RC, Schoeller DA, Kushner R, Hnilicka J, Gold ST (1991) Total daily energy expenditure and activity level in anorexia nervosa. Am J Clin Nutr 53: 1143–1150. 1850575 10.1093/ajcn/53.5.1143

[21] Hall JF, Smith K, Schnitzer SB, Hanford PV (1953) Elevation of activity level in the rat following transition from ad libitum to restricted feeding. J Comp Physiol Psychol 46: 429–433. 13109066 10.1037/h0062565

[22] Routtenberg A, Kuznesof AW (1967) Self-starvation of rats living in activity wheels on a restricted feeding schedule. J Comp Physiol Psychol 64: 414–421. 6082873 10.1037/h0025205

[23] Nicholls DE, Viner RM (2009) Childhood Risk Factors for Lifetime Anorexia Nervosa by Age 30 Years in a National Birth Cohort. Journal of the American Academy of Child & Adolescent Psychiatry 48: 791–799. 19564797 10.1097/CHI.0b013e3181ab8b75

[24] Gelegen C, van den Heuvel J, Collier DA, Campbell IC, Oppelaar H, et al (2008) Dopaminergic and brain-derived neurotrophic factor signalling in inbred mice exposed to a restricted feeding schedule. Genes Brain Behav 7: 552–559. 18363853 10.1111/j.1601-183X.2008.00394.x

[25] Kas MJ, van Dijk G, Scheurink AJ, Adan RA (2003) Agouti-related protein prevents self-starvation. Mol Psychiatry 8: 235–240. 12610657 10.1038/sj.mp.4001206

[26] Davis C, Kaptein S, Kaplan AS, Olmsted MP, Woodside DB (1998) Obsessionality in anorexia nervosa: the moderating influence of exercise. Psychosom Med 60: 192–197. 9560869 10.1097/00006842-199803000-00015

[27] Holtkamp K, Hebebrand J, Herpertz-Dahlmann B (2004) The contribution of anxiety and food restriction on physical activity levels in acute anorexia nervosa. Int J Eat Disord 36: 163–171. 15282686 10.1002/eat.20035

[28] Gelegen C, Pjetri E, Campbell IC, Collier DA, Oppelaar H, et al (2010) Chromosomal mapping of excessive physical activity in mice in response to a restricted feeding schedule. Eur Neuropsychopharmacol 20: 317–326. 19896807 10.1016/j.euroneuro.2009.10.001

[29] Klein DA, Mayer LE, Schebendach JE, Walsh BT (2007) Physical activity and cortisol in anorexia nervosa. Psychoneuroendocrinology 32: 539–547. 17462830 10.1016/j.psyneuen.2007.03.007

[30] Festing MFW (1977) Wheel activity in 26 strains of mouse. Laboratory Animals 11: 257–258. 926756 10.1258/002367777780936530

[31] Kelly SA, Nehrenberg DL, Peirce JL, Hua K, Steffy BM, et al (2010) Genetic architecture of voluntary exercise in an advanced intercross line of mice. Physiological Genomics 42: 190–200. 20388837 10.1152/physiolgenomics.00028.2010 PMC3032284

[32] Lightfoot JT, Turner MJ, Daves M, Vordermark A, Kleeberger SR (2004) Genetic influence on daily wheel running activity level. Physiological Genomics 19: 270–276. 15383638 10.1152/physiolgenomics.00125.2004

[33] Lightfoot JT, Leamy L, Pomp D, Turner MJ, Fodor AA, et al (2010) Strain screen and haplotype association mapping of wheel running in inbred mouse strains. Journal of Applied Physiology 109: 623–634. 20538847 10.1152/japplphysiol.00525.2010 PMC2944645

[34] Swallow JG, Garland T, Carter PA, Zhan W-Z, Sieck GC (1998) Effects of voluntary activity and genetic selection on aerobic capacity in house mice (Mus domesticus). Journal of Applied Physiology 84: 69–76. 9451619 10.1152/jappl.1998.84.1.69

[35] Eriksson M, Rasmussen F, Tynelius P (2006) Genetic Factors in Physical Activity and the Equal Environment Assumption – the Swedish Young Male Twins Study. Behavior Genetics 36: 238–247. 16502139 10.1007/s10519-005-9018-7

[36] Joosen AM, Gielen M, Vlietinck R, Westerterp KR (2005) Genetic analysis of physical activity in twins. The American Journal of Clinical Nutrition 82: 1253–1259. 16332658 10.1093/ajcn/82.6.1253

[37] Hofstetter JR, Trofatter JA, Kernek KL, Nurnberger JI, Mayeda AR (2003) New quantitative trait loci for the genetic variance in circadian period of locomotor activity between inbred strains of mice. J Biol Rhythms 18: 450–462. 14667146 10.1177/0748730403259468

[38] Kas MJ, de Mooij-van Malsen JG, de Krom M, van Gassen KL, van Lith HA, et al (2009) High-resolution genetic mapping of mammalian motor activity levels in mice. Genes Brain Behav 8: 13–22. 18721260 10.1111/j.1601-183X.2008.00435.x

[39] Pjetri E, Adan RA, Herzog H, de Haas R, Oppelaar H, et al (2012) NPY receptor subtype specification for behavioral adaptive strategies during limited food access. Genes Brain Behav 11: 105–112. 21923762 10.1111/j.1601-183X.2011.00732.x

[40] Bouten CV, van Marken Lichtenbelt WD, Westerterp KR (1996) Body mass index and daily physical activity in anorexia nervosa. Med Sci Sports Exerc 28: 967–973. 8871905 10.1097/00005768-199608000-00005

[41] Reed DR, Bachmanov AA, Tordoff MG (2007) Forty mouse strain survey of body composition. Physiol Behav 91: 593–600. 17493645 10.1016/j.physbeh.2007.03.026 PMC2085171

[42] Coners H, Remschmidt H, Hebebrand J (1999) The relationship between premorbid body weight, weight loss, and weight at referral in adolescent patients with anorexia nervosa. Int J Eat Disord 26: 171–178. 10422606 10.1002/(sici)1098-108x(199909)26:2<171::aid-eat6>3.0.co;2-p

[43] Steinhausen HC, Grigoroiu-Serbanescu M, Boyadjieva S, Neumarker KJ, Metzke CW (2009) The relevance of body weight in the medium-term to long-term course of adolescent anorexia nervosa. Findings from a multisite study. Int J Eat Disord 42: 19–25. 18683885 10.1002/eat.20577

[44] Svenson KL, Von Smith R, Magnani PA, Suetin HR, Paigen B, et al (2007) Multiple trait measurements in 43 inbred mouse strains capture the phenotypic diversity characteristic of human populations. J Appl Physiol 102: 2369–2378. 17317875 10.1152/japplphysiol.01077.2006

